# Occupational risk of overweight and obesity: an analysis of the Australian Health Survey

**DOI:** 10.1186/1745-6673-5-14

**Published:** 2010-06-16

**Authors:** Margaret A Allman-Farinelli, Tien Chey, Dafna Merom, Adrian E Bauman

**Affiliations:** 1 School of Molecular Bioscience, University of Sydney. NSW 2006 Australia; 2 School of Public Health, University of Sydney. NSW 2006 Australia

## Abstract

**Background:**

Adults spend about one third of their day at work and occupation may be a risk factor for obesity because of associated socioeconomic and behavioral factors such as physical activity and sedentary time. The aim of this study was to examine body mass index (BMI) and prevalence of overweight and obesity by occupation and explore the contributions of socioeconomic factors and lifestyle behaviors (including leisure time and commuting physical activity, diet, smoking, and alcohol) to occupational risk.

**Methods:**

Secondary analyses of the National Health Survey in Australia (2005) were conducted for working age adults (20 to 64 years). Linear and logistic regression models using BMI as either dichotomous or continuous response were computed for occupation type. Model 1 was age-adjusted, Model 2 adjusted for age and socioeconomic variables and Model 3 adjusted for age, socioeconomic variables and lifestyle behaviours. All models were stratified by gender.

**Results:**

Age-adjusted data indicated that men in associate professional (OR 1.34, 95% CI 1.10-1.63) and intermediate production and transport (OR 1.24 95% CI 1.03-1.50) occupations had a higher risk of BMI ≥ 25 kg/m^2 ^than those without occupation, and women in professional (OR 0.71, 95% CI 0.61-0.82), management (OR 0.72, 95% CI 0.56-0.92) and advanced clerical and service occupations (OR 0.73 95% CI 0.58-0.93) had a lower risk. After adjustment for socioeconomic factors no occupational group had an increased risk but for males, professionals, tradesmen, laborers and elementary clerical workers had a lower risk as did female associate professionals and intermediate clerical workers. Adjustment for lifestyle factors explained the lower risk in the female professional and associate professionals but failed to account for the lower odds ratios in the other occupations.

**Conclusions:**

The pattern of overweight and obesity among occupations differs by gender. Healthy lifestyle behaviors appear to protect females in professional and associate professional occupations from overweight. For high-risk occupations lifestyle modification could be included in workplace health promotion programs. Further investigation of gender-specific occupational behaviors and additional lifestyle behaviors to those assessed in the current Australian Health Survey, is indicated.

## Background

The global epidemic of obesity continues to worsen and the ready availability of cheap energy-dense foods and increasing sedentary lifestyle are considered likely causes [1]. There have also been changes in the types of occupation in which workers are employed - from 'high activity' to 'low activity' occupations and the work environment that contemporary workers experience within a given occupation may now involve more sedentary times than previously [2].

Adults of working age may spend as much as 50% of their waking hours in the work environment. Average time spent at work for full time employees is approximately 40 to 46 hours per week in countries such as Australia, the US and Europe [3-5]. Thus occupational physical activity is a potential determinant of total daily energy expenditure. Professional and white collar workers have been shown to take less steps (measured by pedometer) and have lower volumes of occupational physical activity (METmin per week) than blue collar workers [6,7]. Additionally, the type of food available in the work environment may contribute to daily energy consumption.

Considerable emphasis has been placed on the benefits of leisure time physical activity (LTPA) to counteract sedentary occupations and lifestyles but we have previously reported that leisure time activity may be unlikely to contribute sufficient energy expenditure to prevent increases in the prevalence of overweight and obesity [8,9]. Diet and other personal lifestyle behaviors influencing body weight such as walking for transport, alcohol consumption and smoking are rarely considered simultaneously with occupation. Analysis of the BMI of working age adults by occupational group together with lifestyle behaviors would locate occupations with a high prevalence of overweight and obesity and identify potentially modifiable factors to prevent and treat obesity. Such a workforce analysis could be used to select occupational groups requiring intervention and plan risk factor modification interventions for workplaces.

The Australian Bureau of Statistics (ABS) conducts National Health Surveys (NHS) that include information about occupation and working hours as well as BMI, a range of health-related behaviors such as LTPA, walking for transport and socioeconomic factors [10]. The aim of this study was to conduct secondary analyses of the recent NHS to examine differences in BMI and prevalence of overweight and obesity by occupation. Furthermore, a range of socioeconomic and lifestyle behaviors would be explored to determine which if any appeared protective against occupational risk of overweight and obesity.

## Methods

### Data source

The NHS are a series of cross-sectional surveys designed to obtain representative and national benchmark information on a range of health-related issues and to enable the monitoring of trends over time. For these analyses the most recent NHS conducted in 2004/05 was used. The survey used a stratified multistage area sample design from which a sample of private dwellings in urban and rural areas was randomly selected. Information was collected through face-to-face interview with one selected adult. To account for possible seasonal effects on health characteristics, the sample in the survey was allocated equally to each quarter of the calendar year. In total 25,900 people were surveyed with a good response rate of 89.4%. In these analyses data on 14,618 adults (7466 males and 7152 females) was included. Access to the NHS data was obtained through the ABS Confidential Unit Record Files provided on compact disks.

### Occupation and socioeconomic variables

All data were collected by self-report. Information sought from the subjects included their age in years, country of birth, marital status, education (highest level post school), gross weekly income, and occupation. The ABS classifies occupation according to the ten occupational groups used in the national Census. These are managers and administrators, professionals, associate professionals, tradespersons, elementary clerical, sales and service workers, intermediate clerical, sales and service workers, advanced clerical and service workers, intermediate production and transport workers, labourers and those without occupation [10].

### Lifestyle behaviours

Subjects were asked to report their height in cm and weight in kg. The respondents were asked about LTPA in the prior two weeks including number of times spent in exercise in three categories: walking, moderate and vigorous activities. The two-week recall method has been demonstrated to have good repeatability and acceptable validity [11,12]. Respondents were also asked whether they had walked the previous day for periods of ten minutes or more for the purpose of transport, how many times and the total time walked.

Dietary data collected included information on type of milk consumed (whether whole or fat-reduced category) and the number of serves of vegetables and fruit usually consumed each day. Respondents were asked about the types and quantities of alcoholic drinks consumed on the three most recent days in the week prior to the interview when alcohol was consumed. Questions about smoking included whether they currently smoked or ever smoked at least 100 cigarettes and information about ages they had started and ceased smoking.

### Data handling and statistical analysis

The weighting factors for each record were computed by the ABS to reflect the population at the time of the survey, taking into account the probability of being sampled and the differential response across the population.

This analysis was restricted to those aged 20 to 64 years and with reported height and weight. Body mass index (BMI) was computed as weight (kg)/height (m)^2^. Descriptive statistics (weighted by the normalized person weight) for mean BMI (sd) and prevalence of population overweight (BMI ≥ 25 kg/m^2^) and obesity (BMI ≥ 30 kg/m^2^) were tabulated by occupation type, social and economic demographics and health behaviour risk factors.

Differences in BMI and prevalence of overweight and obesity by occupation type were investigated by logistic and linear regression models using BMI measure as dichotomous and as normally distributed continuous response. The differences were modelled by simple and multiple regressions adjusting for age, lifestyle behavior factors, and socioeconomic variables. Model 1 was age-adjusted only, Model 2 included age and socioeconomic variables and Model 3 included age, socioeconomic and lifestyle behaviour variables. Analyses were stratified by gender.

Occupation type (10 categories) was the main variable of interest. Other significant variables for adjustment were: age (3 categories 20-34 years, 35-49 years and 50-64 years), country of birth (COB, 2 categories Australia/English speaking or other) marital status (2 categories married/defacto or other), education level (4 categories school only, basic vocational, diploma or degree), and household income quintile. Health-risk behaviours included physical activity category (LTPA and transport walking, LTPA but no transport, no LTPA but transport walking and no LTPA and no transport), good diet (dichotomised on basis of consuming 2 fruit plus more than 3 vegetable servings daily and using low fat milk or not), alcohol intake (4 categories from abstinence through recommended limits to increased and excessive), and smoking status (2 categories current or not).

Results for overweight and obesity were presented as percentage prevalence and odds ratios with 95% confidence limits and BMI as mean (sd) and regression beta coefficients. All analyses were conducted using SAS (version 9.1, 2002-3; SAS Institute Inc., Cary, NC, USA).

## Results

Table 1 shows the descriptive statistics for mean (sd) BMI and prevalence of overweight and obesity by occupation type and socioeconomic variables. This univariate analysis of the variables shows that with referent to those 'without occupation', male occupational groups with significantly lower mean BMI are the professionals, tradespersons, elementary clerical sales and service workers and laborers. For females, significantly lower mean BMI is found in managers and administrators, professional and associate professional, advanced clerical and service workers and intermediate and elementary sales and service workers. Both the mean BMI and the prevalence of overweight and obesity appear to increase with age for both sexes. Socioeconomic variables associated with significantly higher mean BMI are being born in Australia and English speaking countries, being in a marriage or defacto relationship and having lower education. For the variable of 'household income' (HHI) the results varied between males and females. Lower BMI in males is found with 2^nd ^highest quintile HHI and in females with the lowest quintile HHI.

**Table 1:** Weight status by occupation and sociodemographic variables for the representative Australian population aged 20-64 years.

	Male				Female			
Socio-demographic	n	BMI mean (sd)	Over-weight %	Obese %	n	BMI mean (sd)	Over-weight %	Obese %
Occupation type								
Without occupation	1121	27.1 (4.8)^R^	38.6	25.1	2300	26.0 (5.9)^R^	28.0	20.7
Manager & administrator	852	27.3 (4.4)	49.3	19.3	313	24.9 (4.8)^b^	27.1	13.4
Professional	1100	26.4 (4.2)^b^	45.2	15.6	1162	24.9 (5.0)^b^	23.0	14.7
Associate professional	808	27.3 (4.9)	45.9	23.1	656	25.2 (4.8)^b^	29.1	14.9
Tradesperson	1262	26.6 (4.5)^a^	46.2	17.7	99	25.9 (5.8)	28.6	16.5
Advanced clerical & SW^1^	44	28.3 (5.4)	50.4	27.3	339	25.0 (5.1)^b^	23.5	15.4
Intermediate clerical	526	27.0 (5.0)	45.6	19.9	1314	25.4 (5.4)^b^	27.0	16.9
SSW^2^								
Intermediate production & transport	867	27.4 (4.7)	43.0	24.3	101	25.6 (5.2)	25.7	23.1
Elementary clerical SSW	305	25.8 (4.9)^b^	39.7	14.7	498	25.4 (5.2)^a^	29.4	16.3
Laborers	528	26.3 (4.8)^b^	36.1	22.0	336	25.6 (5.1)	28.5	17.9
Age group								
20-34	2639	25.8 (4.8)^R^	38.6	14.7	2510	24.3 (5.5)^R^	22.4	12.8
35-49	2689	27.4 (4.6)^b^	46.9	23.1	2628	25.6 (5.4) b	27.1	17.6
50-64	2139	27.5 (4.4)^b^	46.8	24.2	2014	26.7 (5.1) b	32.4	23.0
Country of birth								
Australia/English-speaking	6190	27.1 (4.6)^R^	45.0	21.5	5890	25.7 (5.5)^R^	27.2	18.8
Others	1276	26.0 (4.9)^b^	38.6	15.1	1262	24.3 (5.2)^b^	26.0	10.9
Social marital status								
Married/Defacto	4531	27.5 (4.8)^R^	47.9	23.0	4437	25.7 (5.6)^R^	28.7	18.0
Other	2935	25.9 (4.4)^b^	37.9	16.4	2715	25.0 (5.1)^b^	24.1	16.5
Highest level post-school								
None/still at school	2878	27.0 (4.9)^R^	42.9	22.3	3104	26.0 (5.7)^R^	9.4	20.8
Basic/skilled vocational	2261	27.2 (4.6)	45.0	22.4	1381	25.8 (5.6)	26.7	18.9
Diploma	827	27.1 (5.0)	44.7	20.5	1068	25.2 (5.1)^b^	27.3	15.5
Degree or higher	1500	26.1 (4.1)^b^	43.8	13.8	1600	24.2 (4.6)^b^	22.2	10.9
Household income quintile								
1st Quintile (highest)	608	26.9 (5.5)^R^	40.6	20.5	1217	25.9 (6.0)^R^	28.6	20.1
2nd Quintile	664	26.4 (4.8)^a^	34.7	22.4	1127	26.1 (5.6)^b^	29.3	19.9
3rd Quintile	1121	26.7 (4.8)	40.5	19.9	1633	25.4 (5.3)	25.5	17.8
4th Quintile	1747	26.9 (4.7)	43.8	21.2	1266	25.3 (5.1)	28.0	16.4
5th Quintile (lowest)	2626	27.1 (4.2)	49.0	19.7	966	24.9 (4.9)^b^	26.3	13.4

^1 ^SW = Service worker

^2 ^SSW = Sales & service worker

^R ^= Referent group

^a ^= p-value < 0.05

^b ^= p-value < 0.01

Table 2 presents mean BMI and prevalence of overweight and obesity by lifestyle behaviors. LTPA resulted in lower BMI and obesity prevalence for males and any LTPA and/or walking in lower BMI for females. Females who did not drink alcohol demonstrated greater BMI and obesity than those with moderate or large intakes. Females respondents consuming reduced fat milk and adequate serves of fruit and vegetables had higher BMI. Smoking was associated with lower BMI in males.

**Table 2:** Weight status by health behaviours for the representative Australian population aged 20-64 years.

		Male				Female			
Health risk behaviour		n	BMI mean (sd)	Over- weight %	Obese %	n	BMI mean (sd)	Over-weight %	Obese %
Physical Activity category^1^									
No LTPA & No TW		1524	27.4 (5.2)^R^	39.8	25.4	1294	26.2 (6.4)^R^	27.6	22.1
LTPA, No TW		3129	26.8 (4.4)^b^	46.4	18.8	2756	25.4 (5.0)^b^	28.5	16.3
No LTPA, TW		830	27.0 (5.4)	38.1	25.5	839	25.3 (5.9)^b^	27.3	17.3
LTPA & TW		1983	26.6 (4.3)^b^	45.8	17.1	2263	25.1 (5.1)^b^	24.7	16.1
Other Health indicators									
Smoking status									
Not-current		5306	27.1 (4.6)^R^	45.7	21.0	5528	25.5 (5.4)^R^	26.7	17.7
Current		2160	26.3 (4.7)^b^	39.5	19.0	1624	25.2 (5.3)	27.9	16.3
Diet									
Alcohol intake: g/day									
Male: 0	Female: 0	2027	27.0 (5.4)^R^	38.1	23.7	3077	26.0 (6.0)^R^	26.8	21.0
1-40	1-20	3835	26.9 (4.5)	45.8	19.2	2901	25.2 (5.1)^b^	27.1	15.4
41-80	21-60	984	26.6 (4.0)	47.3	17.2	1015	24.8 (4.4)	25.9	13.3
>80	>60	620	27.0 (4.4)	46.3	22.3	160	24.7 (4.6)^a^	34.9	11.2
2 fruit&>3 veg daily^2 ^plus									
Low fat milk		6825	26.9 (4.7)^R^	43.5	20.6	5982	25.3 (5.5)^R^	26.1	16.9
No		641	27.1 (4.3)	48.5	19.1	1171	26.0 (5.0)^b^	31.4	20.1
Yes									

^1 ^LTPA = leisure time physical activity TW = transport-related walking

^2 ^veg = vegetable servings

^R ^= Referent group

^a ^= p-value < 0.05

^b ^= p-value < 0.01

The estimates of BMI by occupational group for each of the three models are shown in Table 3. The age-adjusted BMI coefficients show that professional males, elementary clerical and sales and service workers and laborers have lower BMI than those without occupation but intermediate production and transport workers have a higher BMI. The mean BMI for all males regardless of occupation is above 25 i.e. the overweight category. Females who are managers, professionals, associate professionals and advanced clerical and service workers have lower BMIs than those not working. After adjusting for socioeconomic factors (Model 2) the male professionals and elementary clerical workers still have lower BMI but managers, tradespersons and laborers also have lower BMI and the intermediate transport and production workers no longer have a higher BMI. Female professionals no longer have lower BMI but managers and all levels of clerical and sales and service workers have lower mean BMI. Adjustment for both behavioral and socioeconomic variables (Model 3) produced no further differences between professions.

**Table 3:** Adjusted BMI estimates (se) according to occupation for the representative Australian population aged 20-64 years

	Model 1^1^	Model 2^2^	Model 3^3^
	BMI coefficient (se)	BMI coefficient (se)	BMI coefficient (se)
**Males**			
Without occupation (Intercept, β_o_)	26.0 (0.16)	26.2 (0.19)	27.1 (0.24)
Managers & administrators	0.06 (0.21)	-0.45 (0.23)^a^	-0.47 (0.23)^a^
Professionals	-0.54 (0.19)^b^	-0.6 (0.23)^b^	-0.64 (0.23)^b^
Associate professionals	0.34 (0.21)	-0.12 (0.23)	-0.11 (0.23)
Tradespersons	-0.21 (0.19)	-0.83 (0.21)^b^	-0.84 (0.21)^b^
Advanced clerical & SW^4^	1.20 (0.70)	0.74 (0.7)	0.77 (0.70)
Intermediate clerical SSW^5^	0.11 (0.24)	-0.35 (0.26)	-0.30 (0.26)
Intermediate production & transport	0.43 (0.21)^a^	-0.09 (0.22)	-0.11 (0.22)
Elementary clerical SSW	-0.85 (0.30)^b^	-1.07 (0.30)^b^	-1.06 (0.30)^b^
Laborers	-0.62 (0.24)^a^	-0.93 (0.25)^b^	-0.93 (0.25)^b^
			
**Females**			
Without occupation (Intercept, β_o_)	24.7 (0.15)	24.8 (0.19)	26.0 (0.25)
Managers & administrators	-1.08 (0.32)^b^	-1.07 (0.34)^b^	-0.92 (0.34)^b^
Professionals	-0.82 (0.19)^b^	-0.42 (0.24)	-0.32 (0.24)
Associate professionals	-0.51 (0.24)^a^	-0.81 (0.25)^b^	-0.61 (0.25)^a^
Tradespersons	0.23 (0.55)	-0.13 (0.55)	-0.89 (0.54)
Advanced clerical & SW^4^	-0.73 (0.31)^a^	-1.25 (0.32)^b^	-1.11 (0.32)^b^
Intermediate clerical SSW^5^	-0.28 (0.19)	-0.68 (0.21)^b^	-0.57 (0.20)^b^
Intermediate production & transport	-0.07 (0.54)	-0.50 (0.54)	-0.46 (0.54)
Elementary clerical SSW	-0.23 (0.26)	-0.67 (0.27)^a^	-0.55 (0.27)^a^
Laborers	-0.33 (0.31)	-0.54 (0.32)	-0.52 (0.31)

^1 ^Model 1 = Adjusted for age (3 categories)

^2 ^Model 2 = Adjusted for age country of birth, marital status, education level, household income.

^3 ^Model 3 = Adjusted for age and all socioeconomic as in model 2 plus health behaviours i.e. physical activity category, good diet intake, alcohol intake, smoking status

^4 ^SW = Service worker

^5 ^SSW = Sales & service worker

^a ^= p-value < 0.05

^b ^= p-value < 0.01

Table 4 shows the results of multiple logistic regressions modeling BMI ≥ 25 (i.e overweight and obesity) as the binary response variable with 'occupation type' the main covariate of interest for each gender. After adjustment for age (Model 1) males who are associate professionals or intermediate production and transport workers have a higher OR of overweight or obesity while females who are managers, professionals or advanced clerical and service workers are less likely to be overweight or obese than those without occupation. Adjustment for socioeconomic factors meant there was no longer increased likelihood for male associate professional and intermediate production and transport workers to be overweight but managers, tradespersons, elementary clerical workers and laborers had a decreased risk (Model 2). For females it resulted in additional occupational groups having a lower odds i.e. associate professionals and intermediate clerical and sales and service workers. Model 3 adjusts for lifestyle behaviors and socioeconomic factors. For males the only change in OR was that managers had significantly lower odds in addition to the occupations identified in model 2 and for females adjustment meant that professionals and associate professionals no longer had lower OR.

**Table 4:** Adjusted odds of overweight/obesity by occupation for the representative Australian population aged 20-64 years.

	**Model 1**^1^	**Model 2**^2^	**Model 3 **^3^
	OR (95% CI)	OR (95% CI)	OR (95% CI)
Males			
Without occupation (Intercept, β_o_)	Referent	Referent	Referent
Managers & administrators	1.18 (0.98-1.43)	0.82 (0.66-1.02)	0.80 (0.65-1.0)^a^
Professionals	0.94 (0.79-1.12)	0.77 (0.62-0.96)^a^	0.75 (0.6-0.93)^b^
Associate professionals	1.34 (1.10-1.63)^b^	0.98 (0.78-1.22)	0.97 (0.77-1.21)
Tradespersons	1.13 (0.95-1.34)	0.78 (0.63-0.94)^b^	0.77 (0.63-0.94)^b^
Advanced clerical & SW^4^	2.05 (0.99-4.24)	1.50 (0.71-3.15)	1.45 (0.69-3.05)
Intermediate clerical SSW^5^	1.20 (0.96-1.50)	0.89(0.70-1.13)	0.89 (0.70-1.14)
Intermediate production & transport	1.24 (1.03-1.50)^a^	0.89 (0.72-1.10)	0.91 (0.73-1.12)
Elementary clerical SSW	0.81 (0.63-1.06)	0.68 (0.52-0.9)^b^	0.68 (0.52-0.90)^b^
Laborers	0.86 (0.70-1.07)	0.69 (0.54-0.87)^b^	0.70 (0.55-0.88)^b^
			
Females			
Without occupation (Intercept, β_o_)	Referent	Referent	Referent
Managers & administrators	0.72 (0.56-0.92)^b^	0.71 (0.55-0.92)^b^	0.74 (0.57-0.97)^a^
Professionals	0.71 (0.61-0.82)^b^	0.81 (0.67-0.98)^a^	0.83 (0.69-1.01)
Associate professionals	0.93 (0.77-1.10)	0.82 (0.67-0.99)^a^	0.87 (0.71-1.06)
Tradespersons	0.97 (0.65-1.47)	0.88 (0.58-1.33)	0.88 (0.58-1.34)
Advanced clerical & SW^4^	0.73 (0.58-0.93)^b^	0.6 (0.47-0.77)^b^	0.62 (0.48-0.80)^b^
Intermediate clerical SSW^5^	0.92 (0.80-1.06)	0.8(0.68-0.93)^b^	0.83 (0.71-0.97)^a^
Intermediate production & transport	1.11 (0.74-1.66)	0.95 (0.63-1.43)	0.97 (0.64-1.47)
Elementary clerical SSW	1.02 (0.84-1.24)	0.87 (0.70-1.07)	0.90 (0.73-1.11)
Laborers	0.92 (0.73-1.16)	0.84 (0.66-1.06)	0.84 (0.66-1.06)

^1 ^Model 1 = Adjusted for age (3 categories)

^2 ^Model 2 = Adjusted for age country of birth, marital status, education level, household income

^3 ^Model 3 = Adjusted for age and all socioeconomic as in model 2 plus health behaviours i.e. physical activity category, good diet intake, alcohol intake, smoking status

^4 ^SW = Service worker

^5 ^SSW = Sales & service worker

^a ^= p-value < 0.05

^b ^= p-value < 0.01

## Discussion

Males in intermediate transport and production work have a higher BMI but those in professional and elementary clerical and sales and services jobs and in trades and laboring occupations have lower BMI. Females in professional, associate professional, managerial and advanced clerical occupations have lower BMI than those without occupation. Socioeconomic factors such as country of birth, marital status, education and household income explain some of the occupational differences but are not readily modified. It seems that lifestyle-related behavior protected female professionals and associate professional from overweight and obesity. However, in this analysis the measures of lifestyle factors failed to explain the overall protective effect found for some occupations, meaning that the occupation itself may be protective and/or other determinants not assessed in the Australian Health Survey are responsible. Additional measures of physical activity, sedentary behaviors and dietary habits at work, commuting and leisure time are indicated.

Salmon et al previously reported on a National Heart Foundation survey of urban Australians conducted in 1989 [13]. Four categories of occupation were used; professional (managers, professional and associate professionals) skilled (tradespersons, clerical, sales and service workers) and less-skilled (laborers, production workers) workers and homemakers (not in workforce). The prevalence of overweight and obesity amongst Australians was considerably less 15 years ago [14] but the pattern among the occupations appears not to have markedly changed. In their analysis professionals had a lower prevalence of BMI ≥ 25 than those without occupation (homemakers) or less skilled workers.

A study of 603,139 US workers from 1986 through 2002 found that obesity rates were increasing in all occupational groups regardless of race or gender. Forty one categories of occupation were used and motor vehicle operators, other transportation workers, material moving equipment operators and protective service workers had the highest prevalence [15]. This is in agreement with the finding of the current study that male intermediate transport and production workers have a higher prevalence of overweight and obesity. These are occupations that demand the worker sit for long periods with little opportunity for physical activity and the salaries place the workers in a lower socioeconomic group. As shown in the current study the latter predisposes to overweight and obesity.

Higher socioeconomic status has been reported as a protective factor against overweight and obesity in many studies [16]. Differences in social factors, education and income explained the increased risk in intermediate transport and production workers but not the protective effect of the professional and some clerical and service occupations. It has previously been reported that managers, professionals and white collar workers undertake more LTPA that might compensate for less physical activity at work while those in less skilled positions do greater volumes of physical activity at work [13]. Adjustment for lifestyle factors did not change the occupational risk pattern for males but it did account for the lower risk observed for female professionals and associate professionals.

A recent study in the Netherlands measured sitting time at work and during leisure time for a range of occupations and while there were considerable differences in amount of sitting time at work they found no difference during leisure time so that compensation did not occur in their population [17]. Furthermore, two recent studies showed that working adults who engage in physically demanding work [18] or men in blue collar occupations [7] appear to be more active outside work. This suggests that individual compensation of occupational sitting with active leisure time or active occupational work with sedentary leisure time does not necessarily occur. In the current study the male clerical sales and service workers have risks no different to those without occupation yet for females in these same occupations a significantly lower risk was observed. Conversely, females in trades or laborers have a risk no different to those without occupation yet their male counterparts have a lower risk. This divergence of occupational effect by gender suggests that factors other than occupational physical activity level and the socioeconomic and the lifestyle factors measured in the current study, influence the likelihood of overweight and obesity.

Respondents failing to participate in LTPA had higher unadjusted BMI. In a cross-sectional study of 158 middle-aged Australian women it was found that those with no LTPA and most occupational sitting had the lowest number of daily steps and highest BMI [19].

Female respondents with the healthier diet using reduced fat milk and having higher fruit and vegetable consumption, had higher BMI but these crude measures of diet cannot correct for total energy intake and those eating more fruit and vegetables may be eating more food altogether. It is also possible that the overweight respondents may be treating themselves with diet to prevent or treat weight gain but this is impossible to discern from the cross-sectional Health Survey and even cohort studies provide limited evidence that higher fruit and vegetable consumption protects against overweight and obesity [20].

Male smokers have lower BMI as has previously been demonstrated in other studies [21]. Females consuming lower and higher amounts of alcohol had a lower BMI. Females of a higher socioeconomic status are more likely to drink [22]. This is a further demonstration that combinations of behavioral factors confer protection from overweight and obesity even though some behaviors have undesirable consequences for overall health.

There are several limitations of the current study the most obvious being the cross-sectional analytical design describing associations but not causation. In addition, the data are by self-report. It is known that subjects tend to underestimate weight and overestimate height so that BMI values may be higher than calculated whether this differs by occupational group cannot be discerned [23]. The questions concerning LTPA have been demonstrated to have good validity and reproducibility. Assessment of sedentary behaviors at work and at home were not included in this Health Survey and together with direct measures of occupational physical activity, might be important to further explain occupational differences and inter-individual variations within occupations. More complete assessment of dietary intake is also indicated.

## Conclusions

In conclusion, workers involved in intermediate transport and production jobs and women without occupation appear to be most in need of intervention. Changes in occupational, transport and leisure time energy expenditure and in diet are likely to be beneficial for these groups. While socioeconomic status cannot readily be changed, education with respect to healthier lifestyles and about management of food budgets can be offered along with strategies to change behaviors. As the age-adjusted mean BMI of males in all occupations was greater than 25 i.e. overweight, comprehensive workplace health promotion programs for additional occupations should be considered. Professional and associate professional women provide evidence that better lifestyles can lower the risk of overweight and obesity.

## Competing interests

The authors declare that they have no competing interests.

## Authors' contributions

MAF participated in all aspects including conception, study design, analysis, interpretation of results and manuscript writing.

TC participated in the design, statistical analysis, interpretation of results and manuscript writing.

DM participated in all aspects including conception, study design, analysis, interpretation of results and manuscript writing.

AB participated in the study design, interpretation of results and manuscript preparation.

All authors read and approved the final manuscript
